# Suppressing α-1,6-Fucosyltransferase With a Novel Small Molecule Inhibitor Ameliorates Inflammation in Murine T-Cell–Mediated Colitis Models

**DOI:** 10.1016/j.jcmgh.2026.101865

**Published:** 2026-08-14

**Authors:** Akiko Asakura, Shinichiro Shinzaki, Yoshiyuki Manabe, Koki Hizume, Yuri Tsujii, Taku Tashiro, Takahiro Amano, Mizuki Tani, Yuriko Otake-Kasamoto, Takeo Yoshihara, Ryotaro Uema, Yoshiki Tsujii, Hideki Iijima, Yoshihiro Kamada, Daisuke Okuzaki, Daisuke Motooka, Tomohiko Fukuda, Takashi Kudo, Satoru Takahashi, Jianguo Gu, Eiji Miyoshi, Koichi Fukase, Yoshito Hayashi, Tetsuo Takehara

**Affiliations:** 1 Department of Gastroenterology and Hepatology, Graduate School of Medicine, The University of Osaka, Osaka, Japan; 2 Department of Gastroenterology, Hyogo Medical University School of Medicine, Hyogo, Japan; 3 Department of Chemistry, Graduate School of Science, The University of Osaka, Osaka, Japan; 4 Department of Materials Engineering Science, Graduate School of Engineering Science, The University of Osaka, Osaka, Japan; 5 Department of Gastroenterology, Osaka International Medical and Science Center, Osaka Keisatsu Hospital, Osaka, Japan; 6 Department of Advanced Metabolic Hepatology, Graduate School of Medicine, The University of Osaka, Osaka, Japan; 7 NGS Core Facility, Research Institute for Microbial Diseases, The University of Osaka, Osaka, Japan; 8 Division of Regulatory Glycobiology, Institute of Molecular Biomembrane and Glycobiology, Tohoku Medical and Pharmaceutical University, Miyagi, Japan; 9 Laboratory Animal Resource Center in Transborder Medical Research Center, and Department of Anatomy and Embryology, Institute of Medicine, University of Tsukuba, Ibaraki, Japan; 10 Department of Molecular Biochemistry and Clinical Investigation, Graduate School of Medicine, The University of Osaka, Osaka, Japan; 11 Institute for Radiation Sciences, The University of Osaka, Osaka, Japan

**Keywords:** Core Fucosylation, FUT8 Inhibitor, Inflammatory Bowel Disease, Murine Colitis

## Abstract

**Background & Aims:**

Inflammatory bowel disease involves intractable intestinal inflammation often refractory to current pharmacotherapies. We investigated the therapeutic potential of a novel, orally available inhibitor targeting *α*-1,6-fucosyltransferase—the sole glycosyltransferase catalyzing core fucosylation of *N*-glycans in mammals—as an anti-inflammatory agent.

**Methods:**

α-1,6-Fucosyltransferase inhibitor efficacy was evaluated in 2 murine models: a trinitrobenzene sulfonic acid–induced colitis model and a naive CD4^+^ T-cell adoptive transfer model using recombination activating gene 2 (*Rag2*)-deficient mice. Amelioration of trinitrobenzene sulfonic acid–induced colitis by the *α*-1,6-fucosyltransferase inhibitor was also evaluated in *Lck*^*cre*^*Fut8*^*fl/fl*^ mice in which *α*-1,6-fucosyltransferase is conditionally abrogated in T cells. Splenic CD4^+^ T cells from wild-type mice were treated with the *α*-1,6-fucosyltransferase inhibitor to evaluate its effects on cell signaling, cytokine production, and T helper 1, 2, and 17 differentiation. In vivo safety was assessed in wild-type mice.

**Results:**

Oral administration of the *α*-1,6-fucosyltransferase inhibitor effectively decreased T-cell core fucosylation and ameliorated trinitrobenzene sulfonic acid–induced colitis and colitis induced by adoptive T-cell transfer. These clinical effects were accompanied by decreased T helper 1 and 2 cytokine production from CD4^+^ T cells. Trinitrobenzene sulfonic acid–induced colitis was attenuated in *Lck*^*cre*^*Fut8*^*fl/fl*^ mice, demonstrating that the ameliorative effect of *α*-1,6-fucosyltransferase inhibition is directly mediated by T cells. In vitro, the inhibitor suppressed T-cell signal transduction and T helper 1, 2, and 17 cell differentiation. No apparent hepatorenal toxicity or intestinal mucus layer disruption was observed.

**Conclusions:**

*α*-1,6-Fucosyltransferase inhibition modulated T-cell core fucosylation and reduced inflammation in murine colitis models without apparent toxicity, indicating that targeting *α*-1,6-fucosyltransferase may offer a novel therapeutic approach for inflammatory bowel disease by disrupting proinflammatory T-cell responses.

## Graphical abstract

**Figure:**
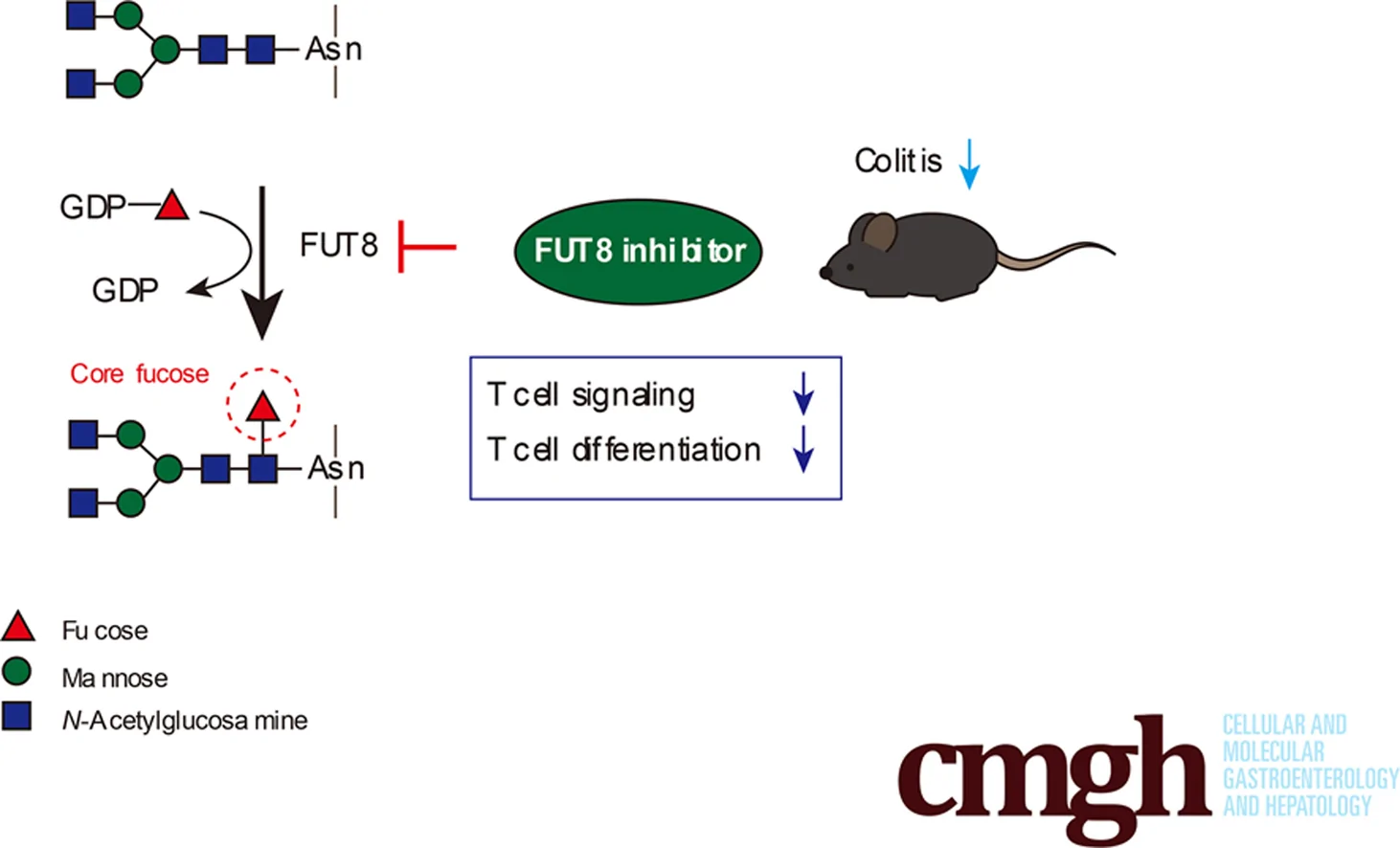

**Summary**

α-1,6-Fucosyltransferase inhibition by a novel small molecule reduces inflammation in murine colitis models by disrupting proinflammatory T-cell responses. Thus, α-1,6-fucosyltransferase is a promising therapeutic target for inflammatory bowel disease.

**What You Need to Know**

**Background**

Developing orally available agents with novel mechanisms is an attractive option for inflammatory bowel disease. We investigated whether targeting α-1,6-fucosyltransferase, the core fucosylation enzyme, ameliorates T-cell–mediated colitis.

**Impact**

Oral administration of a novel α-1,6-fucosyltransferase inhibitor reduced T-cell core fucosylation and suppressed proinflammatory cytokine production, significantly ameliorating intestinal inflammation in murine models of T-cell–dependent colitis.

**Future Directions**

Targeting α-1,6-fucosyltransferase offers a promising therapeutic strategy for inflammatory bowel disease. This orally bioavailable inhibitor that disrupts proinflammatory T-cell responses provides a patient-friendly alternative to injectable biologics.

Inflammatory bowel disease (IBD), principally comprising Crohn’s disease and ulcerative colitis, represents a group of chronic, relapsing inflammatory conditions affecting the gastrointestinal tract. With its global prevalence steadily rising,^1^ this persistent inflammation frequently manifests as severe abdominal pain, chronic diarrhea, and weight loss, profoundly impairing patients’ quality of life and imposing substantial economic burdens on health care systems worldwide.^2^ Despite the availability of various treatment options, including corticosteroids, immunomodulators, and biologics, many patients experience inadequate disease control.^3^^,^^4^ Furthermore, the necessarily parenteral administration of many advanced peptide-based therapies negatively impacts the convenience of patients and their quality of life. Although some orally active treatments have recently emerged, the therapeutic repertoire remains restricted and is often accompanied by considerable safety concerns.^5^ Consequently, novel, orally administered anti-inflammatory agents with a distinct mechanism of action for the effective management of IBD are urgently needed.

We previously demonstrated that serum levels of galactose-deficient IgG are elevated in patients with IBD^6^ and that decreased galactosylation in immune cells improves experimental colitis in mice deficient in *β*-1,4-galactosyltransferase 1 through the interaction of macrophages and B cells.^7^ These findings suggest that the targeted modulation of glycan structures may offer a viable therapeutic strategy for intestinal inflammation. Glycosylation is one of the most important post-translational modifications of proteins and lipids, and the synthetic mechanism is regulated by the activity of characteristic glycosyltransferases during glycan maturation from the endoplasmic reticulum to the Golgi secretory pathway.^8^^,^^9^ This dynamic process confers the structural diversity essential for cellular interactions, with the vast glycome driving the multifunctional capabilities of glycans.^10^ Alterations in cell-surface glycans, governed by the precise activity of distinct glycosyltransferases,^11^ directly dictate the binding affinity of endogenous lectins, thereby modulating cellular crosstalk and downstream signaling.^12^^,^^13^ Glycosylation profiles are highly responsive to genetic and environmental factors and thereby play an instrumental role in modulating immune functions.^8^^,^^14^, ^15^, ^16^ Specifically, glycans critically regulate T-cell development, activation, signal transduction, and differentiation,^14^^,^^17^, ^18^, ^19^ fundamentally impacting T-cell–mediated intestinal immunity. Additionally, fucosylation within the gastrointestinal tract has emerged as a key factor in mucosal host defense against bacterial populations.^20^^,^^21^

Fucosylation, one of the most important modifications of glycans, is broadly categorized into *α*-1,2/1,3/1,4 (Lewis-type), *α*-1,6 (core fucosylation), and *O*-fucosylation, each orchestrating distinct biological processes.^22^ Core fucose is the innermost fucose bound to *N-*acetylglucosamine. The glycome signatures of intestinal T cells from patients with IBD are distinct from those of healthy individuals in that they are characterized by decreased *N*-glycan branching^23^^,^^24^ and elevated core fucosylation.^25^ In mammals, *α*-1,6-fucosyltransferase (FUT8) serves as the sole enzyme responsible for catalyzing core fucosylation.^26^ Our previous research demonstrated that FUT8-deficient (*Fut8*^−/−^) mice are protected from severe colitis and show diminished production of T helper (Th)1 and Th2 cytokines relative to wild-type (WT) controls.^25^ Mechanistically, in T cells from *Fut8*^*−/−*^ mice, the transport of T-cell receptor (TCR)-associated glycoproteins to lipid rafts is inhibited, leading to attenuated downstream TCR signaling.^25^ Although clinical therapeutics targeting glycosyltransferases have yet to be realized,^27^ we recently developed a novel, orally available FUT8 small molecule inhibitor through high-throughput screening and structural optimization (Figure 1*A*).^28^ We hypothesized that this FUT8 inhibitor would exert anti-inflammatory effects by modulating T-cell activity to ameliorate colitis.

**Figure 1:**
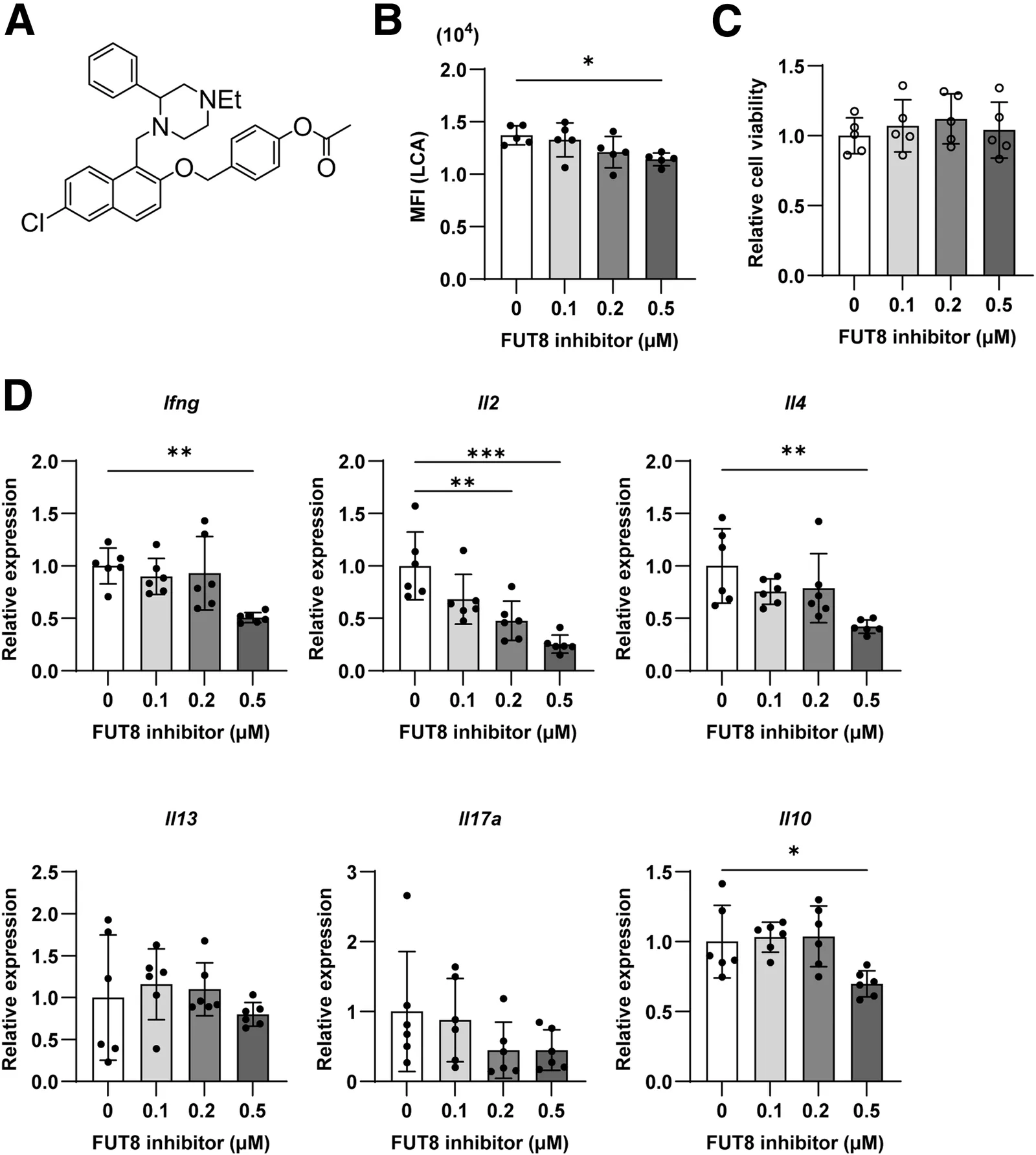
**In vitro effects of the novel FUT8 inhibitor on core fucosylation and cytokine production in murine CD4^+^ T cells.** (*A*) Chemical structure of the novel, orally available FUT8 inhibitor. (*B*) Flow cytometry analysis of cell surface core fucosylation levels. CD4^+^ T cells isolated from murine splenocytes were treated with 0, 0.1, 0.2, or 0.5 μM of the FUT8 inhibitor for 48 hours, and core fucosylation was detected using LCA. (*C*) Cell viability following FUT8 inhibitor treatment. Murine CD4^+^ T cells were incubated with the indicated concentrations of the inhibitor for 48 hours, and viability was assessed to confirm negligible cytotoxicity. (*D*) Cytokine production in activated CD4^+^ T cells. Cells were cultured in vitro and stimulated with anti-CD3ε and anti-CD28 antibodies in the presence of 0, 0.1, 0.2, or 0.5 μM FUT8 inhibitor for 3 hours. Expression levels of cytokine genes were measured by qPCR. Data are presented as the mean ± standard deviation (n = 5 per group for *B* and *C*; n = 6 per group for *D*). ∗ *P* < .05, ∗∗ *P* < .01, ∗∗∗ *P* < .001 vs untreated control (1-way analysis of variance). Similar results were obtained in at least 3 independent experiments. MFI, mean fluorescence intensity.

In the present study, we utilized experimental murine colitis models to evaluate the in vivo efficacy of the FUT8 inhibitor in regulating T-cell responses and mitigating intestinal inflammation, exploring its potential as a novel therapeutic modulator.

## Results

### The Novel α-1,6-Fucosyltransferase Inhibitor Decreases Core Fucosylation and the Production of Inflammatory Cytokines in Murine CD4^+^ T Cells

First, we investigated the levels of core fucosylation on the surface of murine CD4^+^ T cells after treatment with the novel FUT8 inhibitor. The levels of cell surface core fucosylation were analyzed using flow cytometry with *Lens culinaris* agglutinin (LCA), which recognizes *α*-1,6-core fucose. The binding of LCA to CD4^+^ T cells in murine splenocytes was significantly reduced after administration of the FUT8 inhibitor (Figure 1*B*). The cell viability remained unchanged, indicating that the inhibitor exerts negligible cytotoxicity at the applied concentrations (Figure 1*C*). Furthermore, we evaluated the cytokine production in murine CD4^+^ T cells stimulated in vitro with anti-CD3ε and anti-CD28 antibodies. Treatment with the FUT8 inhibitor significantly suppressed the gene expression of Th1- and Th2-related cytokines compared with that of untreated control cells (Figure 1*D*). Collectively, these findings demonstrate that pharmacologic FUT8 inhibition effectively attenuates core fucosylation and the expression of Th1/Th2 cytokines at the messenger RNA level in murine CD4^+^ T cells.

### The α-1,6-Fucosyltransferase Inhibitor Significantly Ameliorates Trinitrobenzene Sulfonic Acid–Induced Colitis

To evaluate the in vivo therapeutic efficacy of the FUT8 inhibitor, we used a murine model of trinitrobenzene sulfonic acid (TNBS)-induced colitis, established by dermal sensitization and subsequent intrarectal TNBS administration. Oral administration of the FUT8 inhibitor significantly attenuated disease severity compared with that in untreated controls, as evidenced by a reduction in body weight loss, colon shortening, and histologic inflammation (Figure 2*A–C*). Furthermore, flow cytometric analysis of the colonic lamina propria revealed a substantial reduction in the absolute numbers of interferon (IFN)-γ^+^ CD4^+^, interleukin (IL)4^+^ CD4^+^, IL17A^+^ CD4^+^, and IL10^+^ CD4^+^ T cells from FUT8 inhibitor-treated mice (Figure 2*D*). Concordantly, the levels of cell surface core fucosylation within the inflamed colon were markedly diminished following treatment (Figure 2*E*). Collectively, these findings demonstrate that the oral administration of this FUT8 inhibitor effectively mitigates TNBS-induced colitis by suppressing core fucosylation and attenuating proinflammatory T-cell responses in the intestinal mucosa.

**Figure 2:**
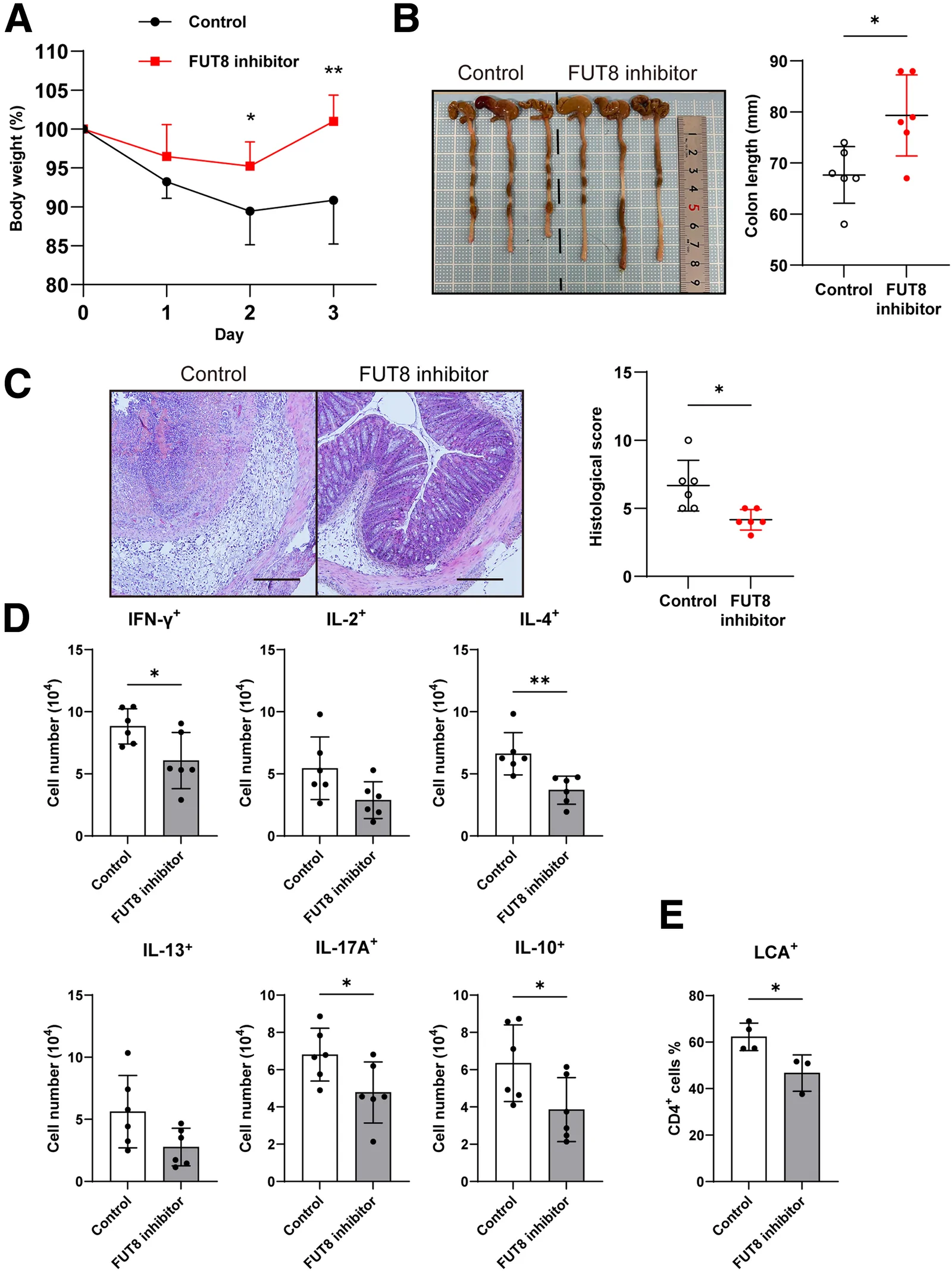
**Oral administration of the FUT8 inhibitor ameliorates TNBS-induced colitis in mice.** C57BL/6J mice were sensitized with 2.5% TNBS by skin painting and challenged 7 days later with intrarectal administration of 2% TNBS to induce colitis. The FUT8 inhibitor (0.5 mg/kg) or vehicle was administered once daily via oral gavage, starting on the day of the intrarectal challenge. (*A*) Body weight changes monitored over the course of the experiment. (*B*) *Left*: Representative images of the colon. *Right*: Colon length on day 3 after TNBS administration. (*C*) *Left*: Representative H&E-stained sections of the distal colon. Scale bars, 200 μm. *Right*: Histologic inflammation scores evaluated on day 3 after TNBS administration. This timepoint corresponds to the most severe local damage. (*D*) Flow cytometric analysis of cytokine-producing T cells in the colonic mucosal lamina propria. The numbers of IFN-γ^+^ CD4^+^, IL2^+^ CD4^+^, IL4^+^ CD4^+^, IL13^+^ CD4^+^, IL17A^+^ CD4^+^, and IL10^+^ CD4^+^ T cells were assessed on day 2 after TNBS administration, corresponding to the peak of clinical symptoms and inflammatory cytokine production. (*E*) Cell surface core fucosylation levels in the colon following FUT8 inhibitor treatment, assessed on day 2 after TNBS administration. Data are presented as the mean ± standard deviation (n = 6 mice per group for *A–D*; n = 3–4 mice per group for *E*). ∗*P* < .05, ∗∗*P* < .01 vs the vehicle-treated TNBS group (2-tailed unpaired Student *t* test). Representative images are from 1 of at least 3 independent experiments with similar findings.

### The α-1,6-Fucosyltransferase Inhibitor Significantly Ameliorates Colitis in an Adoptive T-Cell Transfer Model

To further investigate the effects of the FUT8 inhibitor on the T-cell–mediated induction of murine colitis, a chronic colitis model was established by adoptively transferring naive CD4^+^ T cells into recombination activating gene 2-deficient (*Rag2*^−/−^) mice. Mice treated with the FUT8 inhibitor exhibited significantly reduced body weight loss and colon shortening compared with control mice (Figure 3*A* and *B*). Furthermore, histologic analysis revealed that the FUT8 inhibitor significantly ameliorated colonic inflammation (Figure 3*C*). Coinciding with attenuated disease severity, flow cytometric analyses showed marked reductions in the absolute numbers of IFN-γ^+^ CD4^+^, IL4^+^ CD4^+^, IL13^+^ CD4^+^, and IL17A^+^ CD4^+^ T cells in the colonic lamina propria of FUT8 inhibitor–treated mice (Figure 3*D*). These results suggest that the novel FUT8 inhibitor ameliorates T-cell–dependent colitis, accompanied by a reduction in immunopathologic T-cell responses in the intestinal lamina propria.

**Figure 3:**
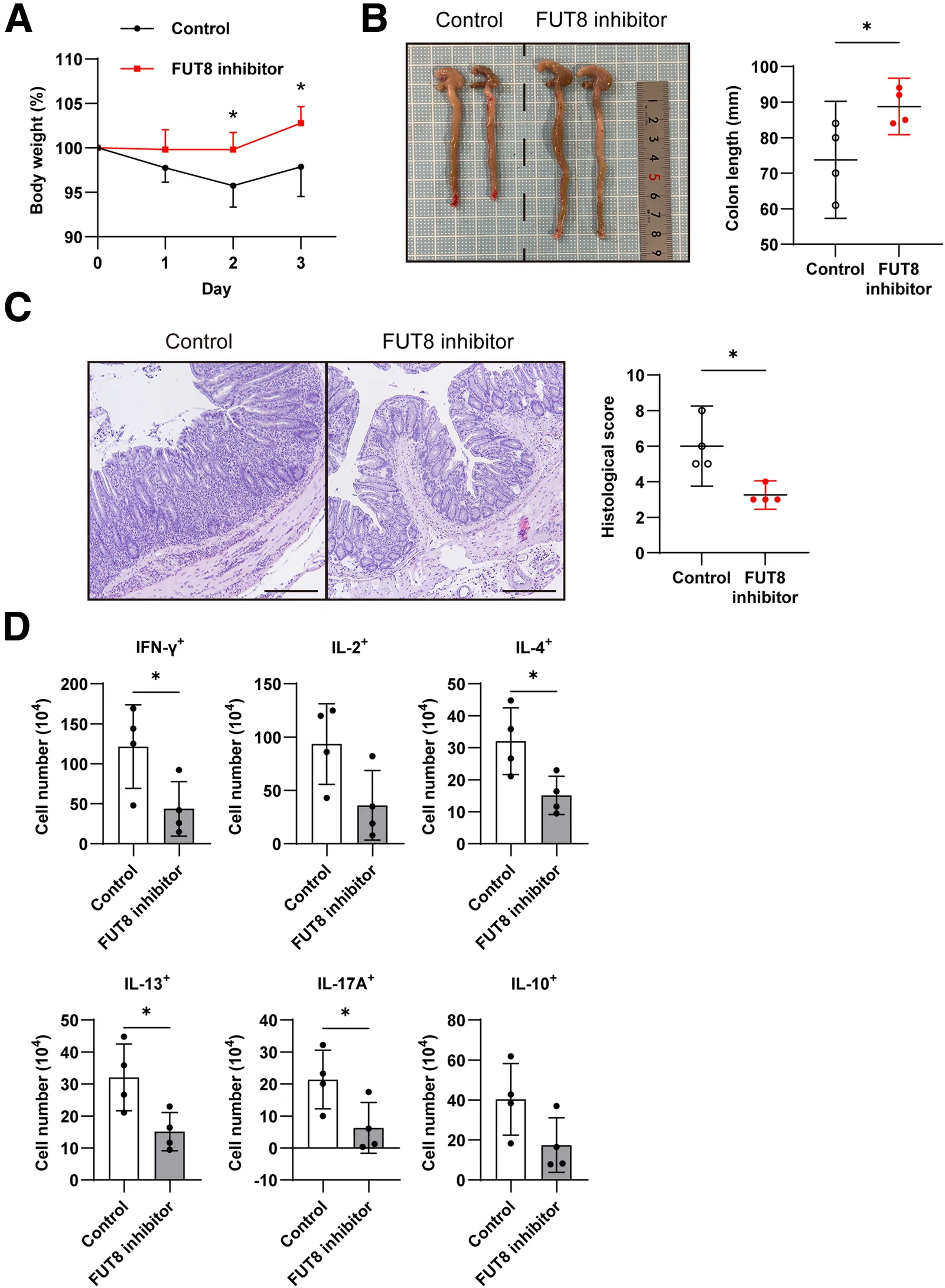
**The FUT8 inhibitor ameliorates T-cell–dependent intestinal inflammation.** T-cell transfer colitis was induced by the intraperitoneal injection of naive CD4^+^ T cells into recombination activating gene 2-deficient (*Rag*2^−/−^) mice. Sixteen days after the transfer, mice received the FUT8 inhibitor (0.5 mg/kg) or a vehicle control once daily via oral gavage for 3 consecutive days. All assessments were performed 24 hours after the final administration. (*A*) Body weight changes in control and FUT8 inhibitor–treated mice over the treatment period. (*B*) *Left*: Representative images of the colon. *Right*: Colon length. (*C*) *Left*: Representative H&E-stained distal colon sections. Scale bars, 200 μm. *Right*: Histologic inflammation scores. (*D*) Number of CD4^+^ T cells in the colonic lamina propria assessed by flow cytometry. Data are presented as the mean ± standard deviation (n = 4 mice per group). ∗*P* < .05 vs the control group (2-tailed unpaired Student *t* test). Representative images are from 1 of at least 3 independent experiments with similar findings.

### Trinitrobenzene Sulfonic Acid–Induced Colitis Is Ameliorated in T-Cell–Specific *Fut8* Conditional Knockout Mice

To elucidate the cellular mechanisms underlying the FUT8 inhibitor–induced decreases in colitis severity and inflammatory cytokine production, we investigated whether these effects are mediated through the modulation of T-cell function. For this purpose, we generated conditional *Fut8* knockout mice (*Lck*^*cre*^*Fut8*^*fl/fl*^ mice), in which the *Fut8* gene is selectively disrupted in the T-cell lineage, and evaluated their susceptibility to TNBS-induced colitis. Flow cytometric analysis confirmed that the levels of cell surface core fucosylation on splenic CD4^+^ T cells were significantly decreased in *Lck*^cre^*Fut8*^fl/fl^ mice compared with those in their *Fut8*^*fl/fl*^ littermates (Figure 4*A*). When subjected to TNBS administration, *Lck*^*cre*^*Fut8*^*fl/fl*^ mice exhibited significantly attenuated disease severity relative to their *Fut8*^*fl/fl*^ littermates, characterized by substantially reduced body weight loss (Figure 4*B*), colon shortening (Figure 4*C*), and histologic colonic inflammation (Figure 4*D*). Consistently, the expression levels of Th1-, Th2-, and Th17-associated inflammatory cytokines were significantly downregulated in *Lck*^*cre*^*Fut8*^*fl/fl*^ mice (Figure 4*E*). To establish that the in vivo protective effect of the pharmacologic FUT8 inhibitor is achieved by targeting FUT8 within T cells, we administered the FUT8 inhibitor to *Lck*^*cre*^*Fut8*^*fl/fl*^ mice with TNBS-induced colitis. Notably, the administration of the FUT8 inhibitor did not confer any additional protection against colitis in *Lck*^*cre*^*Fut8*^*fl/fl*^ mice, showing no further improvement in body weight, colon length, or histologic damage (Figure 4*B–D*). These results suggest that the ameliorative and anti-inflammatory effects of the FUT8 inhibitor in experimental colitis are primarily mediated through the inhibition of FUT8 specifically within T cells.

**Figure 4:**
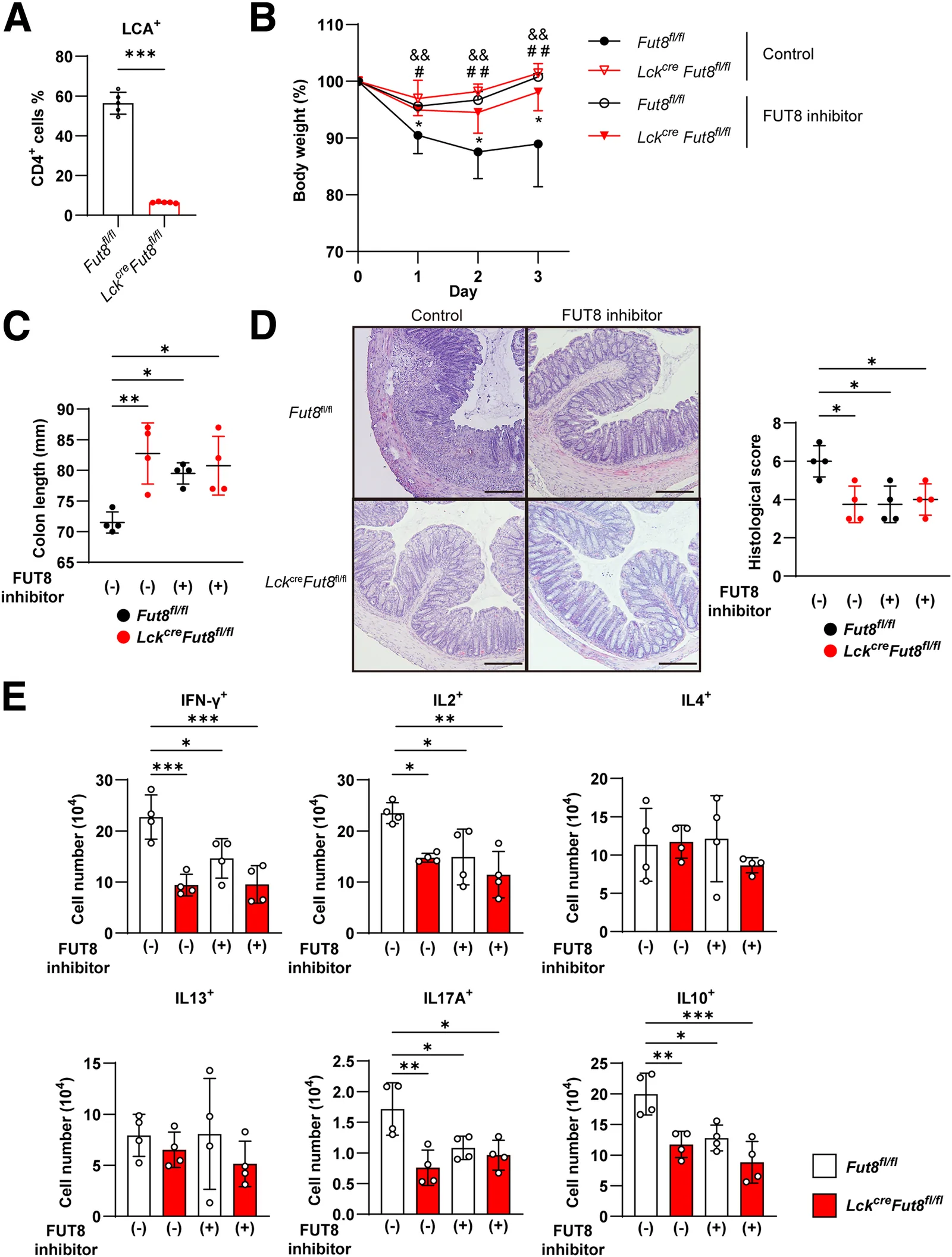
***Lck*^*cre*^*Fut8*^*fl/fl*^ mice exhibit amelioration of TNBS colitis.***Lck*^*cre*^*Fut8*^*fl/fl*^ mice and *Fut8*^*fl/fl*^ littermates were sensitized with 2.5% TNBS by skin painting and received intrarectal 2% TNBS 7 days later to induce colitis. Where indicated, the FUT8 inhibitor (0.5 mg/kg) was administered once daily via oral gavage, starting on the day of the intrarectal administration. (*A*) Flow cytometric analysis of cell surface core fucosylation levels in splenic CD4^+^ T cells isolated from *Lck*^*cre*^*Fut8*^*fl/fl*^ and *Fut8*^*fl/fl*^ mice. The proportion of LCA-positive cells was evaluated. Data are presented as the mean ± standard deviation (n = 5 mice per group). ∗∗∗*P* < .005 vs *Fut8*^*fl/fl*^ littermates (2-tailed unpaired Student *t* test). (*B*) Body weight changes monitored over the course of the experiment. Data are presented as the mean ± standard deviation (n = 4 mice per group). Statistical analysis was performed via 1-way analysis of variance. ^&&^*P* < .01, ^#^*P* < .05, ^##^*P* < .01, ∗ *P* < .05. ^&^*P* denotes a significant difference between *Lck*^*cre*^*Fut8*^*fl/fl*^ and *Fut8*^*fl/fl*^ mice; ^#^*P* denotes a significant difference between *Fut8*^*fl/fl*^ and FUT8 inhibitor-treated *Fut8*^*fl/fl*^ mice, and ∗*P* denotes a significant difference between FUT8 inhibitor-treated *Fut8*^*fl/fl*^ and FUT8 inhibitor-treated *Lck*^*cre*^*Fut8*^*fl/fl*^ mice. (*C*) Colon length measured on day 3 after TNBS administration. Data are presented as the mean ± standard deviation (n = 4 mice per group). Statistical analysis was performed via 1-way analysis of variance. ∗*P* < .05, ∗∗*P* < .01. (*D*) *Left*: Representative H&E-stained distal colon sections evaluated on day 3 after TNBS administration. Scale bars, 200 μm. *Right*: Histologic inflammation scores. Statistical analysis was performed via 1-way analysis of variance. ∗*P* < .05. (*E*) The number of CD4^+^ T cells in the colonic mucosal lamina propria assessed by flow cytometry on day 2 after TNBS administration. Data are presented as the mean ± standard deviation (n = 4 mice per group). Statistical analysis was performed via 1-way analysis of variance. ∗*P* < .05, ∗∗*P* < .01, ∗∗∗*P* < .001.

### T Cells Isolated From *Lck*^*cre*^*Fut8*^*fl/fl*^ Mice Induce Significantly Less Severe Colitis Than Those Isolated From *Fut8*^*fl/fl*^ Mice in the Adoptive T-Cell Transfer Model

To further investigate the specific role of FUT8 in T cells during the induction of murine colitis, we adoptively transferred naive CD4^+^ T cells isolated from either *Lck*^*cre*^*Fut8*^*fl/fl*^ or *Fut8*^*fl/fl*^ mice into immunodeficient *Rag2*^*−/−*^ recipients. Compared with mice receiving naive CD4^+^ T cells from *Fut8*^*fl/fl*^ mice, *Rag2*^*−/−*^ mice engrafted with naive CD4^+^ T cells from *Lck*^*cre*^*Fut8*^*fl/fl*^ mice exhibited significant protection against disease progression, showing less weight loss, reduced colon shortening, and less severe histologic features of colitis (Figure 5*A–C*). Furthermore, the colonic expression of inflammatory cytokines was significantly diminished in *Rag2*^*−/−*^ mice transferred with *Lck*^*cre*^*Fut8*^*fl/fl*^ naive CD4^+^ T cells compared with that in the control group (Figure 5*D*). Collectively, these in vivo results suggest that the ameliorative effect of FUT8 inhibition on colitis is fundamentally based on a T-cell–mediated mechanism.

**Figure 5:**
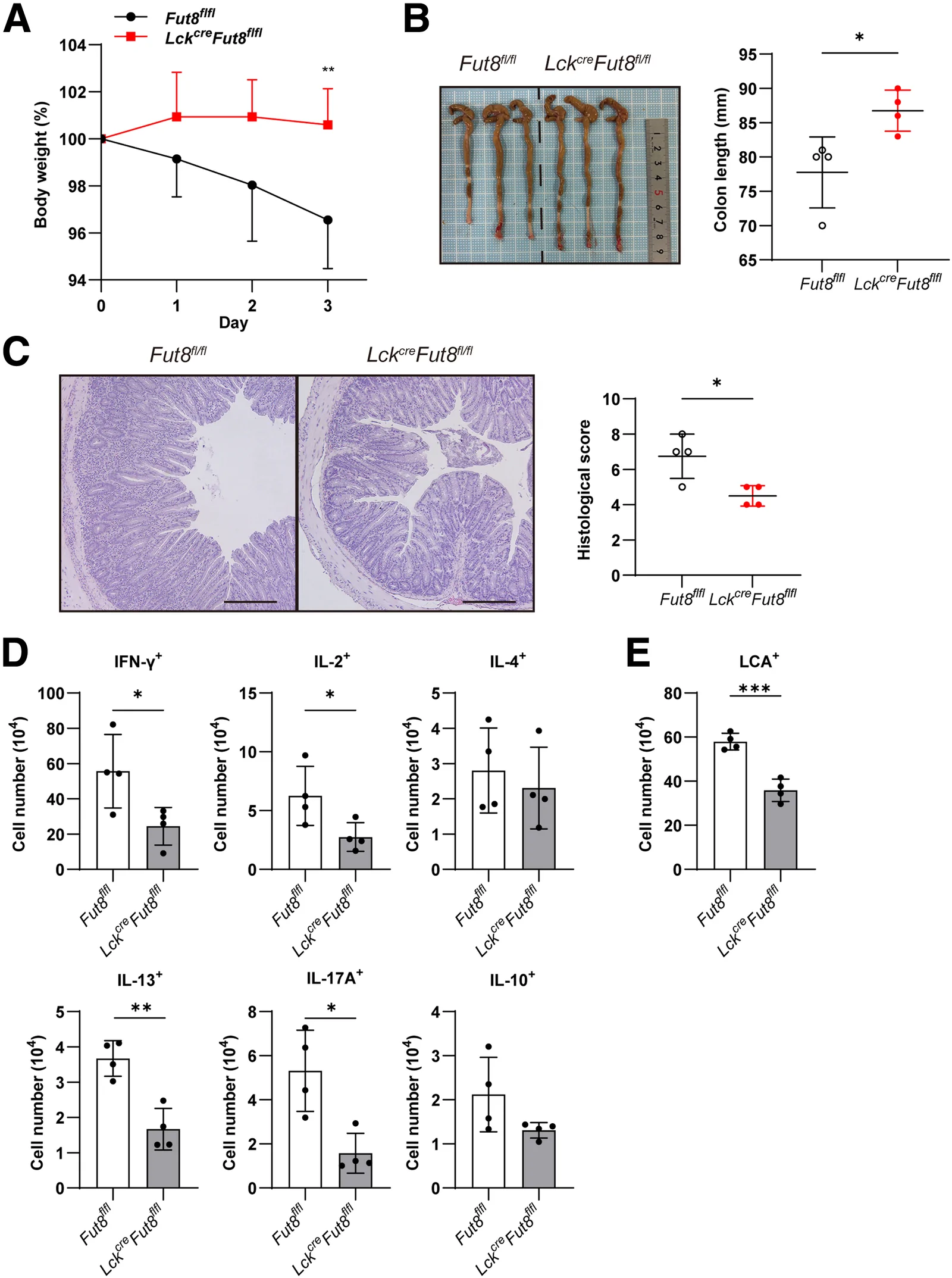
**Adoptive transfer of T cells isolated from *Lck*^*cre*^*Fut8*^*fl/fl*^ mice induces significantly less severe colitis than that of T cells from *Fut8*^*fl/fl*^ mice.** To generate the adoptive T-cell transfer colitis model, naive CD4^+^ T cells isolated from *Lck*^*cre*^*Fut8*^*fl/fl*^ or *Fut8*^*fl/fl*^ mice were transferred into *Rag2*^−/−^ mice via intraperitoneal injection. All assessments were performed on day 16 after the transfer. (*A*) Body weight changes, monitored over the course of the experiment, in *Rag2*^−/−^ mice adoptively transferred with naive CD4^+^ T cells. (*B*) *Left*: Representative macroscopic images of the colon. *Right*: Colon length. (*C*) *Left*: Representative H&E-stained distal colon sections. Scale bars, 200 μm. *Right*: Histologic inflammation scores. (*D*) The number of CD4^+^ T cells in the colonic mucosal lamina propria assessed by flow cytometry. (*E*) Flow cytometric analysis of cell surface core fucosylation levels in CD4^+^ T cells from the colonic lamina propria. The proportion of LCA-positive cells was evaluated. Data are presented as the mean ± standard deviation (n = 4 mice per group). ∗*P* < .05, ∗∗*P* < .01, ∗∗∗*P* < .001 between mice transferred with T cells isolated from *Lck*^*cre*^*Fut8*^*fl/fl*^ mice and those transferred with T cells from *Fut8*^*fl/fl*^ mice (2-tailed unpaired Student *t*-test). Representative images are from 1 of at least 3 independent experiments with similar findings.

### The α-1,6-Fucosyltransferase Inhibitor Suppresses T-Cell Signaling and T Helper 1, 2, and 17 Differentiation

Given that the FUT8 inhibitor exerted its anti-inflammatory effects predominantly by targeting T cells, we next sought to elucidate the intracellular molecular mechanisms underlying this suppression of T-cell activation. Because core fucosylation is crucial for proper TCR signal transduction, we evaluated the effects of the FUT8 inhibitor on downstream signaling cascades. Western blot analysis revealed that in vitro treatment with 0.5 μM FUT8 inhibitor markedly reduced the phosphorylation levels of key mitogen-activated protein kinases, specifically p38, JNK, and ERK, in activated murine CD4^+^ T cells (Figure 6*A*). To determine how this attenuation of intracellular signaling translates to changes in Th cell effector functions, we performed in vitro differentiation assays. Naive CD4^+^ T cells isolated from murine splenocytes were cultured under specific Th1-, Th2-, and Th17-polarizing conditions in the presence or absence of the FUT8 inhibitor. Flow cytometric analysis demonstrated that the administration of 0.5 μM FUT8 inhibitor significantly decreased the proportions of CD4^+^ T cells producing their respective lineage-specific cytokines, namely, IFN-*γ*, IL4, and IL17A, compared with control cells (Figure 6*B*). Collectively, these findings indicate that pharmacologic FUT8 inhibition directly impairs the differentiation of naive CD4^+^ T cells into pathogenic Th1, Th2, and Th17 cell effector lineages by suppressing TCR-mediated intracellular signaling pathways.

**Figure 6:**
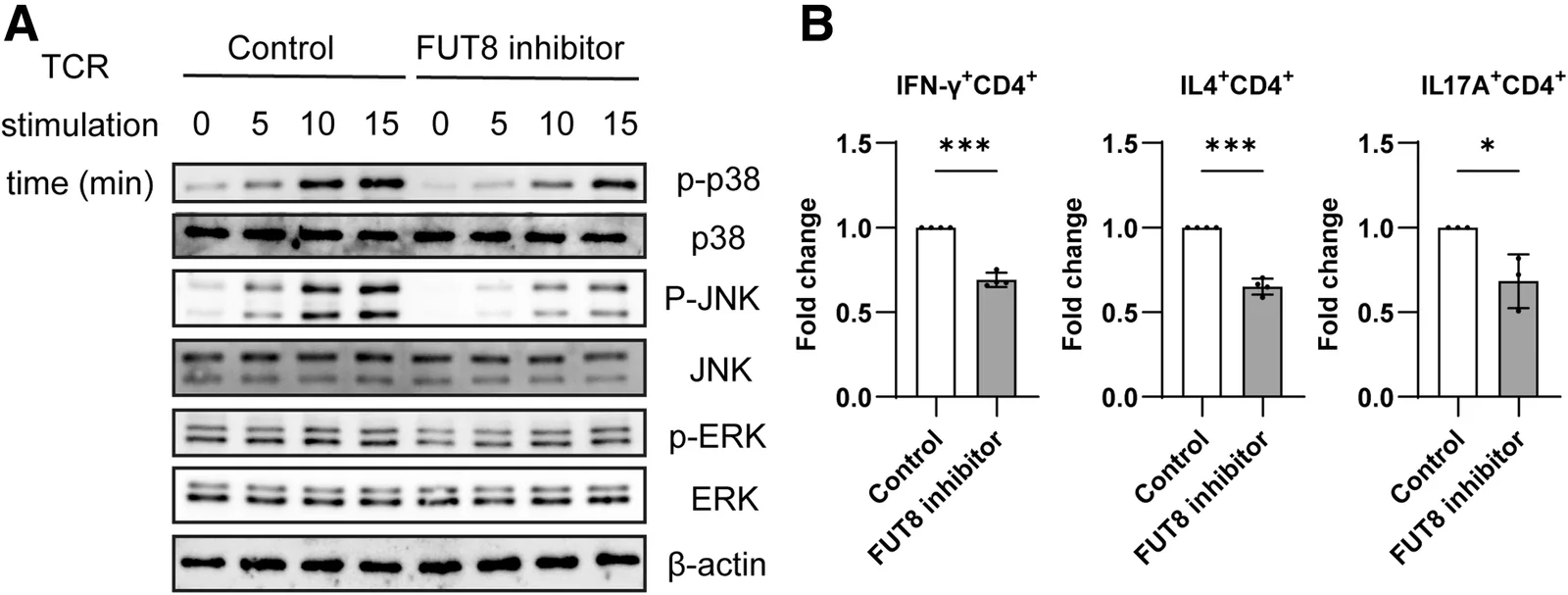
**The FUT8 inhibitor suppresses T-cell signaling and differentiation.** (*A*) Western blot analysis of intracellular signaling molecules in CD4^+^ T cells treated with the FUT8 inhibitor (0.5 μM) for 30 minutes and stimulated with anti-CD3ε and anti-CD28 antibodies. The phosphorylation levels of p38, JNK, and ERK were evaluated. (*B*) Flow cytometric analysis of Th1, Th2, and Th17 cell differentiation. Naive CD4^+^ T cells isolated from murine splenocytes were cultured under respective polarizing conditions and treated with 0.5 μM FUT8 inhibitor for 48 hours. The graph shows the fold-change in the proportion of IFN-γ^+^ CD4^+^, IL4^+^ CD4^+^, and IL17A^+^ CD4^+^ T cells. Data are presented as the mean ± standard deviation (n = 4 for IFN-γ^+^ and IL4^+^ cells; n = 3 for IL17A^+^ cells). ∗*P* < .05, ∗∗∗*P* < .001 vs the vehicle-treated control group (2-tailed unpaired Student *t* test). Representative images are from 1 of at least 3 independent experiments with similar findings.

### Administration of the α-1,6-Fucosyltransferase Inhibitor Shows No Adverse Effects in Mice

To evaluate the potential adverse effects of the FUT8 inhibitor, WT mice received the FUT8 inhibitor via oral gavage for 3 consecutive days. Throughout the administration period, all mice maintained a healthy status, and no significant differences in body weight were observed (Figure 7*A*). In addition, the liver and spleen weights did not differ after administration of the FUT8 inhibitor (Figure 7*B*). To further investigate the potential in vivo hepatotoxicity of the FUT8 inhibitor, the mice were euthanized on the day following the final oral administration, and tissue samples were collected. Hematoxylin and eosin (H&E) staining revealed that the FUT8 inhibitor did not induce hepatic damage, with no abnormalities detected (Figure 7*C*). Furthermore, the serum levels of liver function markers, including alanine aminotransferase and aspartate aminotransferase, as well as those of renal function markers, including blood urea nitrogen and creatinine, remained unchanged in FUT8 inhibitor–treated mice (Figure 7*D*). Histologically, the colon showed no difference in the thickness of the intestinal mucus layer after administration of the FUT8 inhibitor (Figure 7*E* and *F*). Finally, we evaluated the effects of the FUT8 inhibitor on immunologic homeostasis. Quantitative polymerase chain reaction (qPCR) analyses of cells isolated from the spleen after administration of the FUT8 inhibitor showed no differences in the gene expression of inflammatory cytokines (*Ifng*, *Il4*, and *Il17a*) (Figure 7*G*). Similarly, the expression of *Muc2* in intestinal epithelial cells did not differ (Figure 7*H*).

**Figure 7:**
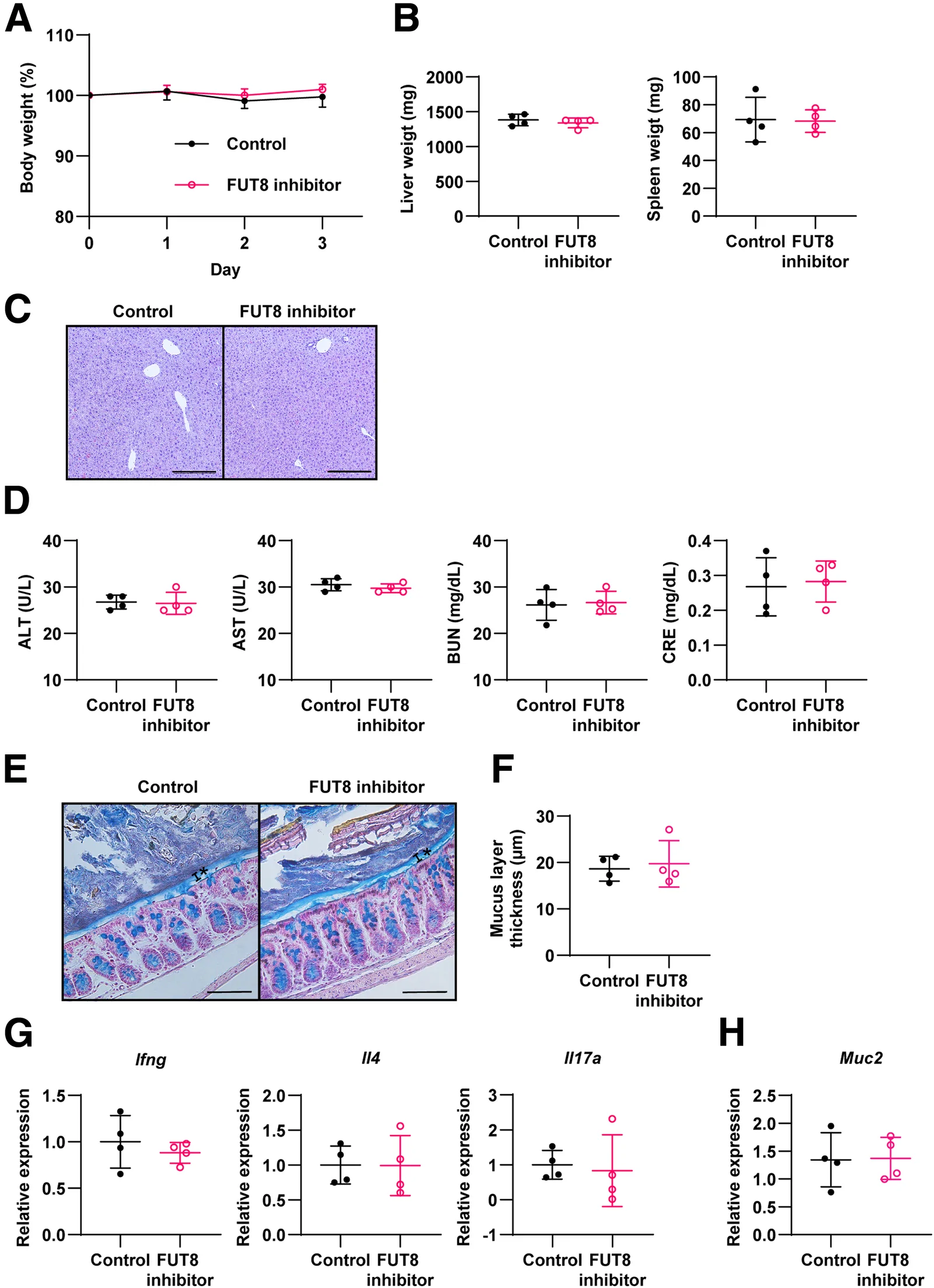
**Safety of the FUT8 inhibitor administration.** Naive male C57BL/6J mice received the FUT8 inhibitor (0.5 mg/kg) or a vehicle control via oral gavage once daily for 3 consecutive days. All assessments were performed 24 hours after the final administration. (*A*) Body weight changes monitored over the course of the experiment. (*B*) Liver and spleen weights. (*C*) Representative H&E-stained images of liver sections. Scale bars, 200 μm. (*D*) Serum levels of alanine aminotransferase (ALT), aspartate aminotransferase (AST), blood urea nitrogen (BUN), and creatinine (CRE) to evaluate hepatic and renal functions. (*E*) Representative images of the mucus layer in the distal colon. Sections were fixed in Carnoy’s solution and stained with Alcian Blue and Nuclear Fast Red. *Stars* indicate the thickness of the inner mucus layer. Scale bars, 200 μm. (*F*) Quantification of the inner mucus layer thickness. (*G*) Relative messenger RNA expression levels of inflammatory cytokines (*Ifng*, *Il4*, and *Il17a*) in cells recovered from the spleen, determined by qPCR. (*H*) Relative messenger RNA expression of *Muc2* in the intestinal epithelium, determined by qPCR. Data are presented as the mean ± standard deviation (n = 4 mice per group). No significant differences were observed between the vehicle-treated and FUT8 inhibitor-treated groups (1-tailed unpaired Student *t* test). Representative images are from 1 of at least 3 independent experiments with similar findings.

## Discussion

The present study demonstrates that the newly developed FUT8 inhibitor substantially ameliorates inflammation in murine models of T-cell–dependent colitis. The current findings extend our earlier observations, which revealed that core fucosylation is markedly elevated at inflammatory sites compared with that at noninflammatory sites in the intestinal tract.^25^ Furthermore, we previously established that *Fut8*^*−/−*^ mice produce lower levels of inflammatory cytokines than WT mice and are significantly protected against T-cell–mediated colitis.^25^ Collectively, these findings strongly suggest that FUT8 represents a promising therapeutic target for IBD.

A previous study demonstrated that colitis is significantly ameliorated in systemic *Fut8*-deficient mice, as well as after the adoptive transfer of *Fut8*-deficient T cells.^25^ In the present study, the novel FUT8 inhibitor significantly reduced the levels of inflammatory cytokines in 2 murine models of T-cell–dependent colitis. Furthermore, our data indicate that in *Lck*^*cre*^*Fut8*^*fl/fl*^ mice, TNBS-induced colitis is significantly ameliorated, accompanied by a reduction in inflammatory cytokines. These results strongly indicate that the FUT8 inhibitor alleviates colitis by modulating T-cell functions. Notably, the effects of the inhibitor are highly consistent with the results observed in the *Lck*^*cre*^*Fut8*^*fl/fl*^ mice used in this study. This concordance strongly supports the mechanistic model wherein the inhibitor, despite systemic administration, exerts its therapeutic efficacy primarily by blocking FUT8 function in T cells.

The previous study also reported that the loss of core fucosylation impairs the translocation of the TCR complex to lipid rafts, thereby suppressing signal transduction.^25^ Mechanistically, lipid rafts are rich in hydrophobic lipids and have a high affinity for hydrophobic molecules. The lack of core fucose—a highly hydrophobic monosaccharide—likely renders TCR-related glycoproteins more hydrophilic, which physically prevents their efficient accumulation in lipid rafts. Consequently, the FUT8 inhibitor prevents the proper assembly of the TCR signaling complex within these microdomains, leading to the suppression of downstream activation and subsequent inflammatory cytokine production.

The treatment of murine splenocytes with the FUT8 inhibitor decreased core fucosylation. Although the number of LCA-positive cells was reduced by only approximately 20%, the ameliorating effect on colitis was comparable to that observed in *Lck*^*cre*^*Fut8*^*fl/fl*^ mice. This indicates that partial suppression of FUT8 is sufficient to alleviate colitis. Moreover, administration of the FUT8 inhibitor to *Lck*^*cre*^*Fut8*^*fl/fl*^ mice did not further ameliorate colitis. A previous study reported that inflammatory cytokine production is suppressed in macrophages from *Fut8*^*−/−*^ mice,^29^ implying that cell types other than T cells might also contribute to the amelioration of colitis by the FUT8 inhibitor. However, our findings from the adoptive transfer colitis model using *Lck*^*cre*^*Fut8*^*fl/fl*^ mice demonstrate that the primary therapeutic action of the FUT8 inhibitor is predominantly mediated through T cells.

A critical prerequisite for any novel oral therapeutic is its safety profile and physiological compatibility. Our in vivo safety evaluations demonstrated that oral administration of the FUT8 inhibitor to naive mice did not induce any apparent hepatotoxicity or systemic adverse effects. Although a previous study has implicated FUT8 in the regulation of secreted mucins in the colon,^21^ our histologic and qPCR analyses indicated that the pharmacologic inhibition of FUT8 did not obviously disrupt the integrity of the intestinal mucus layer or alter *Muc2* expression in naive mice. Likewise, the basal expression levels of inflammatory cytokines in the spleen remained unaffected. These results underscore that, in the absence of an inflammatory trigger, the FUT8 inhibitor does not aberrantly suppress basal immune functions or compromise the mucosal barrier, thereby preserving physiological homeostasis.

Although many existing therapeutic antibodies (such as anti–tumor necrosis factor-α antibodies) are injected, the present inhibitor demonstrated efficacy following oral administration. This route of administration offers a considerable advantage in terms of improving patient quality of life and treatment compliance. The reliance on injectable medications imposes a considerable burden on patients who must integrate these injections into their daily lives. Therefore, the development of orally available therapeutics for IBD is highly attractive, and the orally available FUT8 inhibitor provides a major advantage in the clinical management of IBD. Although genetic ablation represents a congenital deficiency that is essentially prophylactic, our study demonstrated therapeutic efficacy when the drug was administered during the induction or after the onset of inflammation. This highlights the considerable potential of this novel FUT8 inhibitor as a therapeutic agent for the active phase of IBD in actual clinical settings. This study demonstrates that a FUT8 inhibitor, acting as a glycosylation modulator, can serve as an orally available drug candidate for the treatment of IBD, thereby providing a drug discovery approach targeting T-cell glycosylation. Notably, the FUT8 inhibitor offers a distinct mechanism of action compared with current biological therapies. Although conventional biologics, such as anti–tumor necrosis factor-α antibodies, primarily neutralize downstream inflammatory cytokines after they have been produced, the FUT8 inhibitor targets the upstream signaling cascade by modulating glycan modifications on T cells. This unique upstream intervention may provide a crucial alternative for patients with IBD who exhibit primary nonresponse or secondary loss of response to existing biologics.

In summary, we identified a novel FUT8 inhibitor that effectively ameliorated inflammation in murine models of T-cell–mediated colitis. By elucidating the role of FUT8 in intestinal inflammation, we provided compelling evidence to support the use of FUT8 inhibitors as targeted anti-inflammatory agents. Furthermore, the novel FUT8 inhibitor offers a novel mechanism of action and can be administered orally. This provides a considerable clinical advantage, potentially offering a more patient-friendly and cost-effective alternative to the recently developed injectable biologics currently used for IBD treatment.

## Materials and Methods

### Ethical Considerations

Animal experiments were performed in accordance with the Osaka University Guidelines for Animal Experiments and approved by the Animal Experiment Committee of the Osaka University Graduate School of Medicine.

### Mice

C57BL/6J mice were purchased from CLEA Japan. *Rag2*^*−/−*^ mice on C57BL/6J background were purchased from Taconic Biosciences. All experiments were performed using 8- to 12-week-old male mice. Male mice were exclusively used in these experiments to eliminate the potential confounding effects of the female estrous cycle and hormonal fluctuations on immune responses and colitis severity, thereby ensuring data reproducibility. All mice were maintained under specific pathogen-free conditions. *Lck*^*cre*^ mice were purchased from the National Institutes of Biomedical Innovation, Health and Nutrition. *Fut8*^*fl/fl*^ mice were generated on the C57BL/6J background. Genomic DNA covering the *Fut8* gene of C57BL/6J mice was isolated from the RP24-168N15 BAC clone. Two *loxP* sites flanking exon 7 of *Fut8* were introduced using standard restriction enzymes and polymerase chain reaction (PCR)-based cloning techniques. A neomycin-resistance cassette (neo) flanked by flippase recombinase target sites was inserted between exon 7 and exon 8 to prepare a targeting vector. The linearized targeting vector was electroporated into C57BL/6 embryonic stem cells, and G418-resistant clones were selected to obtain the recombinant *Neo* allele. Germline transmission of the *Neo* allele was confirmed using PCR and Southern blot analyses. Male mice chimeric for the targeted allele were mated with female *FLPe*-transgenic mice to remove the *Neo* cassette and generate the floxed *Fut8* allele (*Fut8*^*fl/fl*^). Conditional knockout mice (*Lck*^*cre*^*Fut8*^*fl/fl*^) were generated by crossing *Lck*^*cre*^ with *Fut8*^*fl/fl*^ mice on the C57BL/6J genetic background.

### Colitis Induction

The TNBS-induced colitis model was generated as described previously,^30^ with slight modifications. Briefly, male C57BL/6J mice (8–12 weeks old) were sensitized with 150 μL of 2.5% TNBS in 50% ethanol by skin painting. After 7 days, these mice received an intrarectal administration of 150 μL of 2% TNBS in 50% ethanol. Simultaneously, the FUT8 inhibitor provided by Professor Y. Manabe (#40)^28^ was administered at 0.5 mg/kg once per day via oral gavage using a gavage tube (No.4202; Fuchigami) for 2 to 3 days.

To generate an adoptive transfer colitis model, naive CD4^+^ T cells (5 × 10^5^) isolated from the spleens of C57BL/6J mice using magnetic separation were transferred into *Rag2*^−/−^ mice (8–12 weeks old) via an intraperitoneal injection. After 16 days, the FUT8 inhibitor was administered once per day via oral gavage for 3 days (same dose as in the TNBS-induced colitis model). Mice were euthanized, and the colon was collected 24 hours after the last FUT8 inhibitor administration.

### Sample Collection

In preliminary studies, the most severe clinical symptoms occurred on day 2 after the TNBS administration, when the mice lost the most weight, and the most significant differences were observed between FUT8 inhibitor-treated and control mice. Therefore, we evaluated the presence of inflammatory cytokines 2 days after the intrarectal administration of TNBS. Three days after the intrarectal administration, the most severe local damage to the colon was observed, and H&E staining was performed to investigate structural damage to the colon.

### Histopathologic Analysis

Murine colon and liver specimens were fixed in formaldehyde, embedded in paraffin, and cut into 4-μm–thick sections. These sections were mounted on glass slides and stained with H&E. Each colon was evaluated using an established inflammation score for the TNBS-induced^30^ and adoptive-transfer^31^ colitis models, as described previously.

To optimally preserve and evaluate the intestinal mucin layer, a separate set of colonic segments was maintained with their luminal fecal pellets intact (when present) and immersed in Carnoy’s fixative, as previously described.^32^ After overnight fixation at 4 °C, the tissues were embedded in paraffin and sectioned at a thickness of 4 μm. These sections were then stained with Alcian Blue and Nuclear Fast Red to visualize the mucus layer. All histological images were acquired using a BX53 microscope (Olympus).

### Measurement of Mucus Layer Thickness

Using ImageJ software, the mucus layer thickness was quantified on distal colon sections that had been stained with Alcian Blue and Nuclear Fast Red. For each animal, measurements were taken at 10 distinct locations. Only tissue sections retaining intact fecal pellets were included in this evaluation.

### Induction of T Helper 1–, 2–, and 17–Polarized Cells

Naive CD4^+^ T cells were purified from the spleens of WT C57BL/6, *Lck*^*cre*^*Fut8*^*fl/fl*^, or control littermate (*Fut8*^*fl/fl*^) mice using a naive CD4^+^ T-cell isolation kit (#130-104-453; Miltenyi Biotec), according to the manufacturer’s protocol. Th1, Th2, and Th17 induction was performed using anti-mouse CD3ε antibody (#553057; BD Biosciences), anti-mouse CD28 antibody (#553294; BD Biosciences), anti-mouse IFN-γ antibody (#17-7311-82; eBioscience), anti-mouse IL4 antibody (#16-7041-85; eBioscience), anti-mouse IL2 antibody (#MAB402; R&D Systems), recombinant murine IL12 (#210-12-10UG; Peprotech), recombinant murine IL4 (#214-14-20UG; Peprotech), and recombinant human TGF-β1 (#100-21-10UG; Peprotech). To induce Th1 cells, mouse spleen naive CD4^+^ T cells were incubated in 1 μg/mL anti-CD3ε antibody-coated 24-well plates or 48-well plates in the presence of 10 μg/mL anti-IL4 antibody, 10 ng/mL IL12, and 2 μg/mL anti-CD28 antibody. To induce Th2 cells, mouse spleen naive CD4^+^ T cells were incubated in 1 μg/mL anti-CD3ε antibody-coated 24-well plates or 48-well plates in the presence of 10 μg/mL recombinant murine IL4, 10 ng/mL anti–IFN-γ antibody, and 0.5 μg/mL anti-CD28 antibody. To induce Th17 cells, mouse spleen naive CD4^+^ T cells were incubated in 1 μg/mL anti-CD3ε antibody-coated 24-well plates in the presence of 10 μg/mL anti-IL4 antibody, 10 μg/mL anti–IFN-γ antibody, 1 μg/mL anti-IL2 antibody, 2 μg/mL anti-CD28 antibody, 50 ng/mL IL6, 2 ng/mL TGF-β, 10 ng/mL IL1β, and 20 ng/mL IL23. After 24 and 48 hours, the FUT8 inhibitor was added to the culture, and the cells were incubated for 24 hours.

### Flow Cytometry

To analyze splenic T-cell subsets, CD4^+^ T cells were separated using CD4 magnetic beads (130-117-043; Miltenyi Biotec). For intracellular cytokine staining, the cells were stimulated with 50 ng/mL phorbol myristate acetate (#P8139; Sigma-Aldrich) and 5 μM ionomycin (#I9657; Sigma-Aldrich) at 37 °C for 3.5 hours in the presence of GolgiStop (554715; BD Biosciences). Fixable Viability Stain 780 (565388; BD Biosciences) was added during surface marker staining and used to detect live cells. Fluorochrome-labeled anti-CD4, anti–IFN-γ, anti-IL2, anti-IL4, anti-IL13, anti-IL17A, and anti-IL10 antibodies were purchased from BioLegend (#100434, #505806, #503809, #504117, #159403, #506917, and #505035, respectively), and LCA was purchased from Vector Laboratories (#FL 1041-5). Surface staining was performed with an anti-CD4 antibody at 4 °C for 10 minutes, and intracellular cytokine staining was performed with anti–IFN-γ, anti-IL2, anti-IL4, anti-IL13, anti-IL17A, and anti-IL10 antibodies for 20 minutes using a Cytofix/Cytoperm Kit Plus (#554715; BD Biosciences), according to the manufacturer’s instructions. Flow cytometric analysis was performed using FACSCanto II machines (BD Biosciences) and analyzed using FlowJo software (TreeStar Inc).

### Quantitative Reverse Transcription Polymerase Chain Reaction

Total RNA was extracted using an RNeasyMini Kit (74104; Qiagen), and complementary DNA was synthesized using the ReverTra Ace qPCR RT Master Mix (FSQ-201; Toyobo), according to the manufacturer’s instructions. Real-time reverse transcription PCR was performed with TaqMan Gene Expression Assays (Thermo Fisher Scientific) (Table 1) using the THUNDERBIRD Probe qPCR mix (#QPS-101; Toyobo) on an ABI Prism 7900HT sequence detection system (Applied Biosystems). All values were normalized to the expression of β-actin, and relative expression levels were calculated. The amplification conditions were 50 °C (2 minutes), 95 °C (10 minutes), and 45 cycles of 95 °C (15 seconds) and 60 °C (60 seconds).

**Table 1:** Primer Sets for Reverse Transcription Quantitative Polymerase Chain Reaction

Gene name	Manufacturer
Mouse β-actin	Thermo Fisher Scientific (Mm02619580_g1)
Mouse IFN-γ	Thermo Fisher Scientific (Mm01168134_m1)
Mouse IL2	Thermo Fisher Scientific (Mm00434256_m1)
Mouse IL4	Thermo Fisher Scientific (Mm00445259_m1)
Mouse IL10	Thermo Fisher Scientific (Mm01288386_m1)
Mouse IL13	Thermo Fisher Scientific (Mm00434204_m1)
Mouse IL17A	Thermo Fisher Scientific (Mm00439618_m1)

IFN, interferon; IL, interleukin.

### Western Blot Analysis

The cells were lysed in radioimmunoprecipitation assay buffer containing protease and phosphatase inhibitor cocktails (03969-21 and #07575-51, respectively; Nacalai Tesque) on ice for 30 minutes. Afterward, the solution was centrifuged at 10,000 × *g* for 15 minutes at 4 °C, and the supernatant was collected. Equal amounts of total protein from different samples were electrophoresed on a sodium dodecyl sulfate-polyacrylamide gel. After blocking the membrane with Bullet Blocking One (#03953-95; Nacalai Tesque), the membranes were washed with Tris-buffered saline containing 0.1% Tween-20 and incubated overnight at 4 °C with the primary antibody in Can Get Signal Immunoreaction Enhancer Solution 1 (#NKB-101; Toyobo). The following antibodies were used for immunodetection: anti–p-p38, p38, p-JNK, JNK, p-ERK, ERK, and β-actin (#9211, #9212, #9251, #9252, #4370, #4695, and #4967, respectively; Cell Signaling Technology). After washing with Tris-buffered saline containing Tween-20, the membranes were incubated with the secondary antibody in Signal Immunoreaction Enhancer Solution 2 (NKB-101; Toyobo). SuperSignal West Pico PLUS Chemiluminescent Substrate (34580; Thermo Fisher Scientific) was used for luminescence analysis. The bands were detected, and protein expression levels were analyzed using Fusion Solo S (Vilber Lourmat).

### Statistical Analysis

All statistical analyses were conducted using GraphPad Prism software version 10.2.0 (GraphPad Software Inc). To evaluate significance, a 2-tailed unpaired Student *t* test was applied for comparisons between 2 independent groups. For datasets containing 3 or more groups, a 1-way analysis of variance was performed. Tukey’s test was utilized for multiple pairwise comparisons among all groups, whereas Dunnett’s test was chosen when comparing multiple experimental groups against a single control group. Significance was set at *P* < .05.

## Supplementary Material

Supplementary Material

Extended PDF
