# Supplementary material for: Suppressing α-1,6-Fucosyltransferase With a Novel Small Molecule Inhibitor Ameliorates Inflammation in Murine T-Cell–Mediated Colitis Models
Source: Cell Mol Gastroenterol Hepatol. 2026 Aug 14;20(12):101865. doi: 10.1016/j.jcmgh.2026.101865 (PMC13635695; doi:10.1016/j.jcmgh.2026.101865)
Supplement: Supplementary file 1 [file mmc1.pdf]

Figure 6A

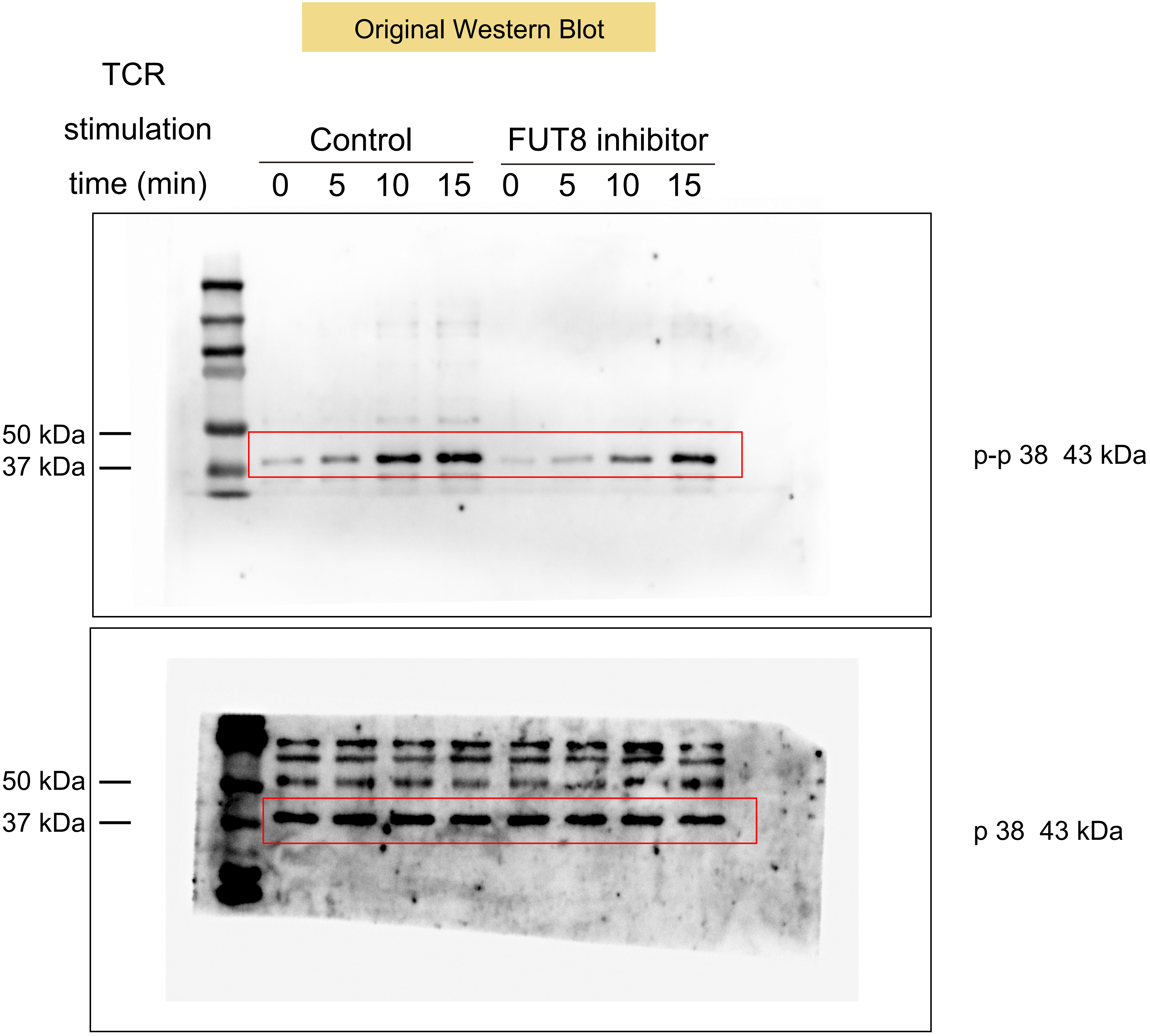
Original Western Blot
TCR
stimulation
time (min)
Control
FUT8 inhibitor
0 5 10 15 0 5 10 15
50 kDa
37 kDa
p-p 38 43 kDa
50 kDa
37 kDa
p 38 43 kDa

Original Western Blot

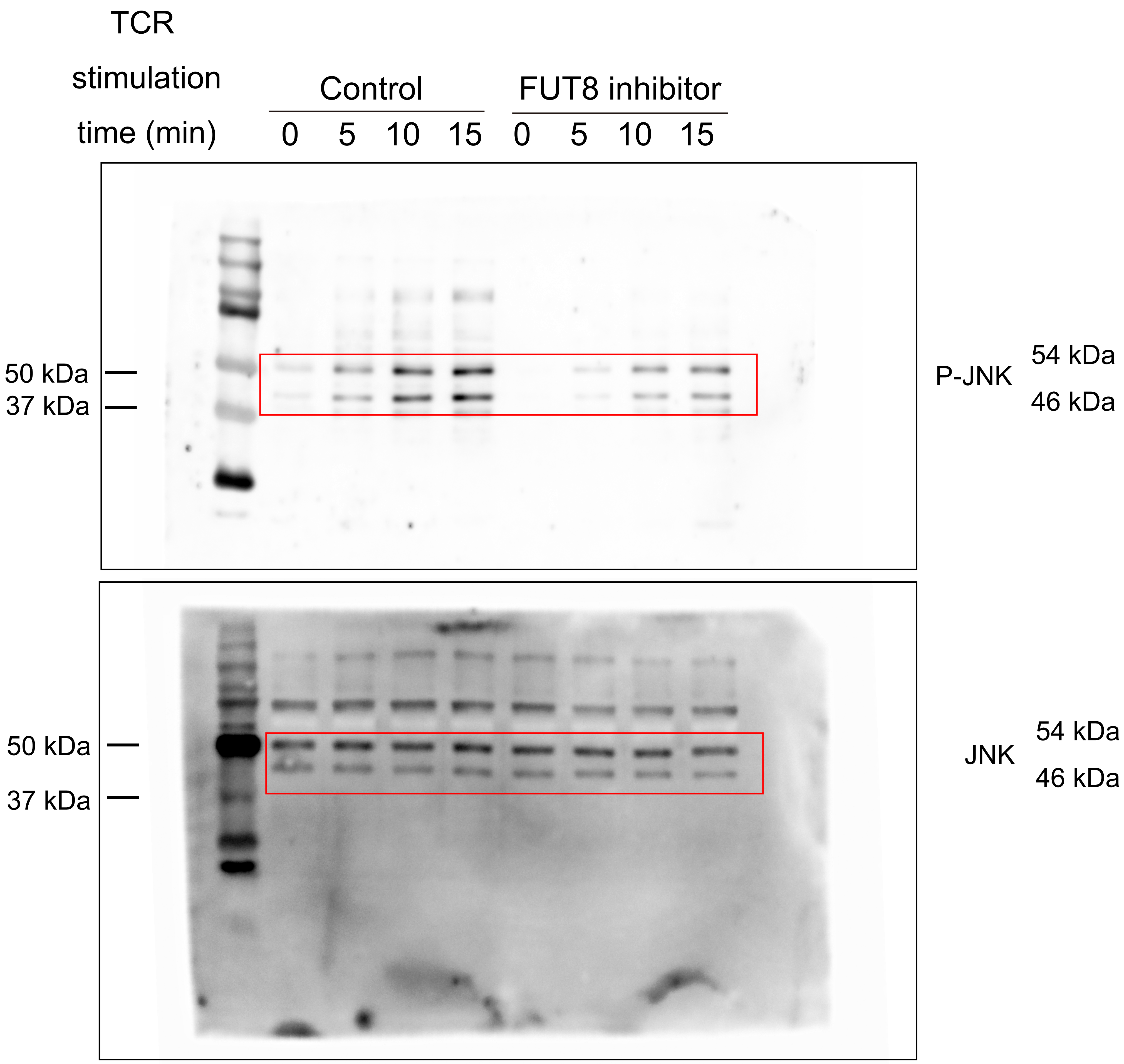

Original Western Blot

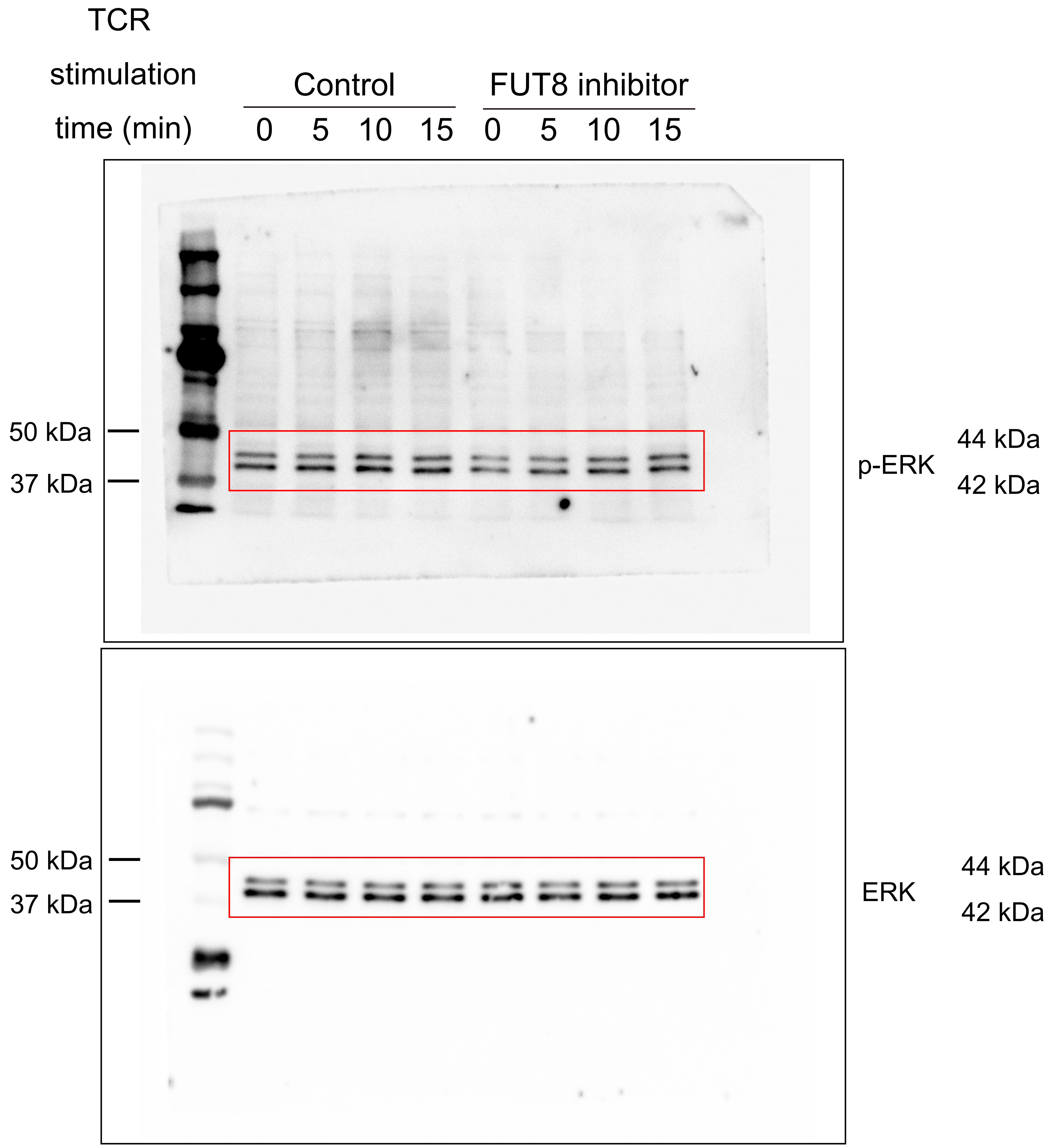

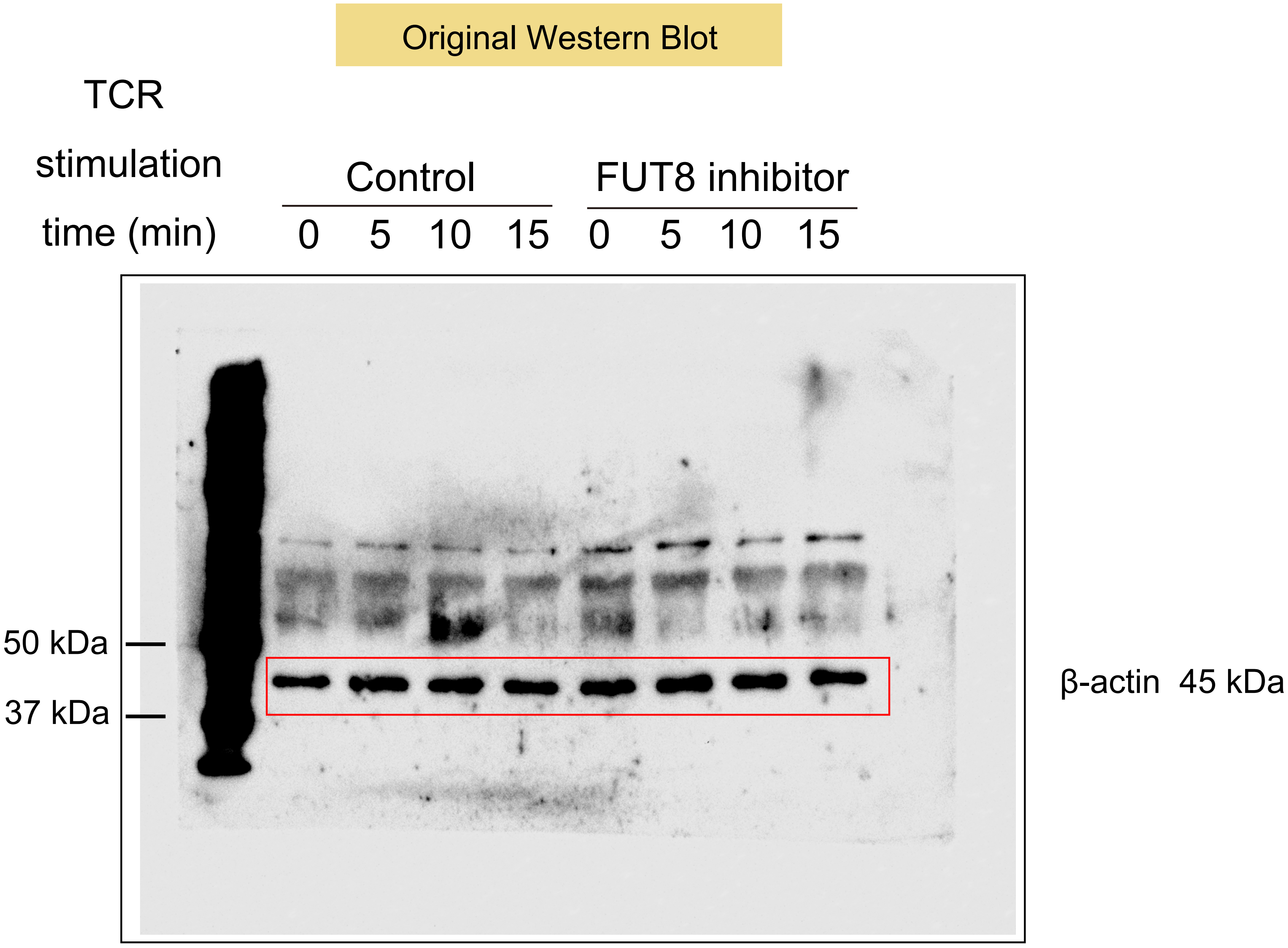
Original Western Blot
TCR
stimulation
time (min)
Control
FUT8 inhibitor
0 5 10 15 0 5 10 15
50 kDa
37 kDa
β-actin 45 kDa
